# USER-CENTRED DIGITAL TRANSFORMATION: JAPANESE OLDER ADULTS’ PERSPECTIVES ON DIGITIZATION

**DOI:** 10.1093/geroni/igae098.3763

**Published:** 2024-12-31

**Authors:** Mineko Wada, Kazuya Takeda

**Affiliations:** Hiroshima University, Hiroshima, Hiroshima, Japan; Kaneda Hospital, Maniwa, Okayama, Japan

## Abstract

This community-based study aimed to explore how Japanese community-dwelling older adults perceived the use of digital technologies in daily life. Although the importance of including older adults in aging and technology research and development has been increasingly emphasized in recent years and the Japanese government has been using the slogan “No One Left Behind” to promote user-centred digital transformation, older adults’ perspectives on a digitized society have not been explored in depth to date. In this qualitative study, 18 Japanese older adults participated in either semi-structured individual interviews (10 participants) or focus groups (8), depending on their preferences. Both the interviews and focus groups elicited participants’ experiences of adapting to digitization in their daily lives and their related perceptions of this shift (i.e., challenges, benefits, and concerns). All the interviews and focus group discussions were digitally recorded, transcribed verbatim, and analyzed thematically. Our analysis generated five themes that illustrate Japanese older adults’ negative and positive perspectives on digital transformation: 1) disapproval of dehumanization, 2) concerns about being monitored and controlled through the digitizing of personal information, 3) increasing risks of scams and fraud, 4) lack of access to immediate support for issues relating to digital adaptation, and 5) potential for expanding social connection and entertainment. We discuss strategies for addressing negative perspectives among older adults that are related to low digital literacy, including, for example, increasing and improving the accessibility of immediate support to help with challenges relating to digitization.

